# Four-year residual impacts of single biochar application on soil fertility and microbial community structure in aeolian sandy soils of semi-arid Inner Mongolia

**DOI:** 10.3389/fmicb.2025.1619992

**Published:** 2025-07-16

**Authors:** Lei Zhou, Junqi Chu, Yufen Zhang, Qi Wang, Yanting Liu, Baoping Zhao

**Affiliations:** ^1^College of Grassland Science, Inner Mongolia Minzu University, Tongliao, China; ^2^College of Life Sciences and Food Engineering, Inner Mongolia Minzu University, Tongliao, China; ^3^Tongliao Agriculture and Animal Husbandry Science Research Institute, Tongliao, China; ^4^Agricultural Public Utilities Service Center, Tongliao, China; ^5^College of Agronomy, Inner Mongolia Agricultural University, Hohhot, China

**Keywords:** biochar, soil fertility, microbial community, buckwheat field, aeolian sandy soil

## Abstract

Biochar (BC) application is widely recognized as a promising strategy for enhancing soil fertility; however, its lasting effects on microbial communities in aeolian sandy soils of semi-arid regions remain poorly understood. To fill this knowledge gap, we conducted a field experiment to evaluate long-term changes in soil properties and microbial community structure in a buckwheat cropping system, 4 years after a single application of biochar (BC) at rates of 0 (BC0), 20 (BC1), 40 (BC2), and 60 (BC3) Mg ha^−1^ in aeolian sandy soils of Inner Mongolia, China. Results revealed significant improvements in soil pH, moisture content, organic carbon (SOC), and available nutrients, as well as microbial biomass and enzyme activity, particularly at higher BC application rates (BC2 and BC3). SOC increased by 9.42% (BC2) and 14.13% (BC3). BC application altered microbial community composition, with minimal effects on bacterial diversity but reduced fungal diversity. Enhanced soil C and N cycling was linked to shifts in key microbial genera, while relative abundances of potential pathogens such as *Fusarium* and *Nothophoma* declined by up to 58 and 77%, respectively. Mantel tests confirmed significant correlations between shifts in microbial diversity and community composition and changes in soil properties, with particularly strong associations for fungal diversity related to SOC (*r* = 0.50, *p* < 0.001) and microbial biomass carbon (SMC; *r* = 0.43, *p* < 0.001). Redundancy analysis further revealed that bacterial communities were significantly associated (*p* < 0.05) with pH, microbial biomass nitrogen (SMN), and invertase activity, while fungal communities were linked to pH, microbial biomass phosphorus (SMP), and urease activity. This study underscores the potential of biochar to enhance soil health by improving soil fertility, reshaping microbial community composition, and suppressing soil-borne pathogens, particularly at higher application rates. These findings provide valuable insights for the reclamation of degraded sandy soils in semi-arid regions on a global scale.

## 1 Introduction

Globally, sandy soils cover an estimated 4.99 × 10^9^ hectares (31% of the terrestrial land area) (Huang and Hartemink, 2020), including approximately 1.7 × 10^8^ hectares in China (Bockheim, 2023). Despite inherent limitations such as poor structure, low nutrient availability, and reduced crop yields, the extensive sandy soils of China are indispensable for sustaining food production (Zhou et al., 2019). Therefore, the effective utilization of sandy soils emerges as a promising strategy for expanding the global area of cultivable land to meet the food needs of a growing population.

The application of organic amendments is increasingly recognized as a promising strategy for improving soil fertility, sustaining soil health, preserving soil multifunctionality, and fostering beneficial effects on plant growth (Diacono and Montemurro, 2010). Biochar (BC) has become widely utilized in agricultural systems to improve soil quality and promote sustainable agricultural practices (Lehmann et al., 2011; Wang et al., 2018; Zhang H. et al., 2021; Antonangelo et al., 2025). This carbon-rich material, produced through anaerobic pyrolysis of biomass, is distinguished by its high carbon content, alkaline properties, and substantial porosity (Lehmann et al., 2011). It also possesses an extensive specific surface area, high cation exchange capacity, efficient nutrient retention capacity, and remarkable structural stability (Wu et al., 2019). Biochar applied to soil can reduce the soil bulk density, increase water holding capacity (Sun et al., 2021), enhance soil carbon, elevate nutrient levels, and improve crop production (Doan et al., 2015). Furthermore, biochar can significantly alter soil microbial biomass, microbial activity, and community composition (Chen et al., 2013; Yang et al., 2025). Despite the key role of BC in soil remediation and enhancement, limited research has been conducted on the effects of long-term biochar application on soil fertility and microbial community composition, particularly in infertile agricultural soils, such as sandy soils. This knowledge gap is especially pronounced in the aeolian sandy soils of the semi-arid regions of northeastern China.

In agro-ecosystems, microorganisms play a crucial role in promoting plant growth, facilitating the mineralization of soil organic matter, driving nutrient cycling, and enhancing soil structure (Hu et al., 2023). The abundance and structure of soil microbial communities have been widely used as indicators of soil quality changes due to their sensitivity to environmental changes (Chen et al., 2013). Biochar directly influences soil microorganisms by altering the soil’s physical and chemical characteristics, such as pH, porosity, and nutrient availability (Chen et al., 2013; Xu et al., 2017). Numerous studies have demonstrated that biochar changed soil microbial diversity and composition, but it varied on biochar properties (Dai et al., 2018; Dominchin et al., 2025; Siddiqui, 2025), soil types (Hu et al., 2014; Chen et al., 2015), application rates (Zhang and Shen, 2022; Hu et al., 2023; Qi et al., 2025), duration (Grossman et al., 2010; Khodadad et al., 2011). So far, limited long-term field studies have resulted in insufficient attention being given to the residual effects of a single biochar application on microbial communities in aeolian sandy soils of semi-arid regions.

The Horqin sandy land, one of the four largest sandy lands in China, encompasses an area of 5.06 × 10^4^ km^2^ in Northeast China (Dai et al., 2018). In this region, Buckwheat (*Fagopyrum esculentum* Moench) is a widely cultivated crop with a long history and extensive area of cultivation, owing to its adaptability to low-fertility soils and cool climatic conditions (Zhou et al., 2024). Given the inherently low fertility, adverse microenvironment, and reduced crop productivity characteristic of aeolian sandy soils, there is an urgent need to develop environmentally sustainable strategies focused on enhancing soil fertility and ameliorating the microenvironment, to promote and sustain long-term soil health (Nepal et al., 2023). To address these knowledge gaps, we conducted a four-year field experiment to investigate the long-term residual effects of biochar application at different rates (0, 20, 40, and 60 Mg ha^−1^) on soil physicochemical and microbial properties of aeolian sandy soil in a semi-arid buckwheat cultivation system. We hypothesized that the application of biochar would improve specific physicochemical properties of aeolian sandy soil while simultaneously influencing microbial α-diversity and community composition. Furthermore, it is anticipated that higher application rates of biochar would result in more persistent effects over time.

## 2 Materials and methods

### 2.1 Site description

The field experiment was at the Xiliaohe village, Tongliao, Inner Mongolia, China (43°44′N, 122°24′E). The region has a temperate continental monsoon climate, with an average annual temperature of 6.4°C, average annual precipitation of 399 mm, and a frost-free period of approximately 150 days. The soil is aeolian sandy soil. The basic properties at a depth of 0–20 cm, prior to the experiment, are: soil organic matter (SOM) = 7.46 g·kg^−1^, available P (AP) = 7.12 mg·kg^−1^, and available K (AK) = 81.44 mg·kg^−1^.

### 2.2 Biochar and field experiment design

Maize stover biochar was obtained from Jinhefu Agriculture Development Company, Liaoning, China. It was produced through slow pyrolysis in a vertical kiln at 450°C for 90 min under oxygen-limited conditions, yielding a 35% conversion of the initial biomass to biochar. The basic properties of the biochar were as follows: pH = 8.85, total carbon (TC) = 657.8 g kg^−1^, total nitrogen (TN) = 9.20 g kg^−1^, total phosphorus (TP) = 8.85 g kg^−1^, total potassium (TK) = 12.3 g kg^−1^, AP = 120 mg kg^−1^, AK = 289 mg kg^−1^, and ash content = 16.02%.

The experiment employed a completely randomized design with four biochar treatments: BC0 (0 Mg ha^−1^), BC1 (20 Mg ha^−1^), BC2 (40 Mg ha^−1^), and BC3 (60 Mg ha^−1^). Initiated in June 2020, biochar was uniformly applied to the upper 15 cm of the soil and thoroughly incorporated using a rototiller to ensure even distribution. Each treatment was replicated three times in plots of 3 m by 10 m. From 2020 to 2023, all plots were annually sown with common buckwheat (cv. Tongqiao No. 5) at a seeding rate of 45 kg ha^−1^ in July, with consistent harvesting occurring in September each year. Prior to sowing each year, all experimental plots received a base fertilizer application of 36 kg ha^−1^ N, 40 kg ha^−1^ P₂O₅, and 81 kg ha^−1^ K_2_O. Field management practices were uniformly applied across all treatments, following local agricultural guidelines.

### 2.3 Soil sampling and analysis

On 13 August 2023, coinciding with the flowering stage of buckwheat. Rhizosphere soil samples were collected by gently shaking off loosely attached soil and using a sterile brush to remove any remaining particles (Fan K. et al., 2020). All visible roots and debris were removed, and the remaining soil was passed through a 2 mm sieve to achieve a homogeneous mixture of the soil samples. To avoid cross-contamination, each replicate from every treatment was sieved using a new, unused sieve. Nine soil samples were collected from each treatment and combined into a single composite sample to ensure representative analysis of each treatment. The composite sample was split into three portions: one was immediately frozen with dry ice and stored at −80°C for DNA analysis; another was stored at 4°C for microbial biomass carbon (SMC), nitrogen (SMN), phosphorus (SMP), and enzyme activity measurements, which were completed within 1 week after sampling; the third was air-dried in the shade at room temperature for 2 weeks to determine soil physicochemical properties.

Soil physicochemical properties were evaluated in accordance with standardized protocols. Specifically, Soil pH was determined with a pH meter using a 1:2.5 (w/v) soil-to-water ratio. Soil water content (SWC) was determined by oven-drying at 105°C for 24 h until a constant weight was achieved. Soil organic carbon (SOC) was quantified by potassium dichromate oxidation (Mebius, 1960), total nitrogen (TN) content was determined using the Kjeldahl method (Barbano et al., 1990), and available phosphorus (AP) was measured by the Olsen method (Olsen et al., 1982). Soil AK content was determined through flame photometry following extraction with ammonium acetate (Helmke and Sparks, 1996). MBC, MBN, and MBP were quantified using the fumigation-extraction method as described in previous studies (Brookes et al., 1982; Brookes et al., 1985). The activities of three extracellular soil enzymes— urease (EC 3.5.1.5), invertase (EC 3.2.1.26), and alkaline phosphatase (EC 3.1.3.1)—were measured using established methods. Urease activity was determined by the sodium phenate–sodium hypochlorite colorimetric method, invertase activity via the 3,5-dinitrosalicylic acid assay, and alkaline phosphatase activity by disodium phenyl phosphate colorimetry, as described by Zhou et al. (2022) and Guan et al. (1991).

### 2.4 DNA extraction and sequencing

Soil DNA was extracted from 0.25 g of soil using the OMEGA Soil DNA Kit (M5636-02; Omega Bio-Tek, Norcross, GA, United States) following the manufacturer’s instructions and stored at −20°C for later analysis. DNA concentration was measured using a NanoDrop NC2000 spectrophotometer (Thermo Fisher Scientific, Waltham, MA, United States), while purity was evaluated through agarose gel electrophoresis. The bacterial community was analyzed by high-throughput sequencing of the 16S rRNA gene, while fungal diversity was assessed via sequencing of the internal transcribed spacer (ITS) region. PCR amplification of the bacterial 16S rRNA gene V3–V4 region was conducted using primers 338F (5′-ACTCCTACGGGAGGCAGCA-3′) and 806R (5′-GGACTACHVGGGTWTCTAAT-3′) (Yu et al., 2005), while the fungal ITS region was amplified with the ITS1F primer set (ITS5-1737F: 5′-GGAAGTAAAAGTCGTAACAAGG-3′ and ITS2-2043R: 5′-GCTGCGTTCTTCATCGATGC-3′) (Degnan and Ochman, 2011). The amplicons were purified, quantified, and combined in equimolar amounts before sequencing on the Illumina NovaSeq platform. The sequencing was performed using the NovaSeq 6000 SP Reagent Kit (500 cycles) at Shanghai Personal Biotechnology Co., Ltd. (Shanghai, China). The raw sequencing data have been archived in the NCBI database under the accession number PRJNA1162243.

### 2.5 Bioinformatic analysis

Sequence data were processed using QIIME2, with adjustments made according to the official guidelines.^1^ Primer sequences were removed with the cutadapt plugin, and quality control, denoising, sequence merging, and chimeric sequence removal were carried out using the DADA2 plugin (Katoh et al., 2002; Callahan et al., 2016). Amplicon sequence variants (ASVs) were inferred based on exact sequence variants using the DADA2 denoising algorithm, which does not involve clustering. Sequences were clustered into ASVs at 100% similarity (Wang et al., 2022). To minimize sampling bias and ensure comparability across samples, rarefaction was performed to normalize sequence depth, with all samples rarefied to the minimum sequencing depth prior to diversity analyses. Bacterial and fungal taxonomic assignments were performed using the SILVA Release 132 and UNITE Release 8.0 databases, respectively (Zheng et al., 2018).

### 2.6 Statistical analysis

Data analysis was conducted using R version 4.2.2,^2^ with visualizations created in both R and Origin Pro 2021 (OriginLab, Northampton, United States). Diversity indices at the ASV level, including Chao1 richness, observed species count, Shannon diversity, and Simpson index, were calculated from the ASV table in QIIME2 and visualized as box plots. Soil property variations, α-diversity, and microbial community composition were assessed through one-way ANOVA. β-diversity, including Permutational Multivariate Analysis of Variance (PERMANOVA) and Principal Coordinates Analysis (PCoA) based on Bray-Curtis dissimilarities, was performed using the ‘vegan’ package in R. Redundancy Analysis (RDA) was used to explore the relationship between microbial composition and environmental variables. Mantel correlations between environmental factors and genus-level taxonomic composition, as well as between environmental variables and α-diversity following BC addition, were computed using the “vegan” package.

## 3 Results

### 3.1 Soil properties

The application of BC significantly (*p* < 0.05) changed the properties of aeolian sandy soil (Table 1). Specifically, the addition of biochar resulted in an increase in soil pH ranging from 0.14 to 0.21 units relative to the untreated soil control. Biochar addition significantly influenced SWC, SOC, AP, and SMC content (*p* < 0.05). These parameters exhibited substantial increases with higher biochar application rates. The BC3 treatment exhibited the most pronounced effects, with increases of 75.62, 14.13, 14.75, and 49.50% for SWC, SOC, AP, and SMC, respectively, compared to BC0. In contrast, the BC1 treatment did not significantly affect TN, AK, SMN content, or invertase activity, while higher biochar rates (BC2 and BC3) led to significant enhancements. Specifically, compared to BC0, the BC2 and BC3 treatments resulted in increases by 14.29 and 19.05% for TN, 8.81 and 7.61% for AK, 16.07 and 17.21% for SMN content, and 6.62 and 8.61% for invertase activity, respectively. However, no significant differences were observed between the BC2 and BC3 treatments. In terms of SMP and alkaline phosphatase activity, biochar application significantly increased both parameters compared to BC0, except for the BC1 treatment (*p* < 0.05). The highest content of SMP was recorded in BC2, while the greatest alkaline phosphatase activity was observed in BC3, with respective increases of 10.93 and 10.76% relative to the control. In addition, biochar application significantly elevated urease activity compared to BC0, with the peak value occurring in BC2 (*p* < 0.05). However, there was no significant difference in urease activity between BC1 and BC3 treatments.

**Table 1:** Effects of different amount of biochar addition on soil physicochemical and microbiological properties.

Treatment	pH	SWC (%)	SOC (g·kg)	TN (g·kg)	AP (mg·kg)	AK (mg·kg)	SMC (mg·kg)	SMN (mg·kg)	SMP (mg·kg)	Urease (mg g ^−1^ 24 h^−1^)	Invertase (mg g ^−1^ 24 h^−1^)	Alkaline phosphatase (μg g^−1^ h^−1^)
BC0	8.04c	8.45d	4.46d	0.42b	7.93d	81.49b	127.79d	7.03b	4.21c	152.89c	1.51b	365.92c
BC1	8.17b	9.5c	4.77c	0.43b	8.77c	83.61b	144.06c	7.19b	4.27c	179.03b	1.5b	369.27c
BC2	8.24a	12.34b	4.88b	0.48a	8.91b	88.67a	169.22b	8.16a	4.67a	188.3a	1.61a	384b
BC3	8.22a	14.84a	5.09a	0.5a	9.1a	87.69a	191.05a	8.24a	4.42b	180.42b	1.64a	405.31a

Different letters in each column represent significant differences between different treatments (*p* < 0.05). BC0, BC1, BC2, and BC3 represent BC application rates at 0, 20, 40, and 60 Mg ha^−1^, respectively. SWC, soil water content; SOC, soil organic carbon; TN, total nitrogen; AP, available phosphorus; AK, available potassium; SMC, SMN, and SMP represent soil microbial biomass carbon, nitrogen, and phosphorus, respectively.

### 3.2 Soil bacterial and fungal community diversity

**Figure 1:**
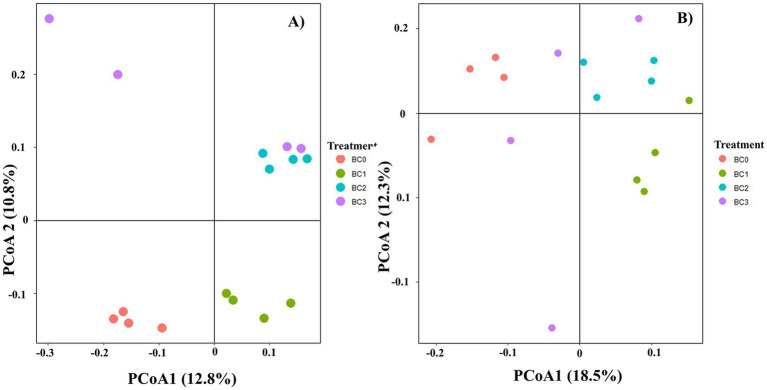
Alpha diversity (α-diversity) of soil bacterial **(A)** and fungal **(B)** communities under different BC treatments. Data are means ± SE (*n* = 4). In each plot, different lowercase letters show statistically significant differences (*p* < 0.05). BC0, BC1, BC2, and BC3 represent BC application rates at 0, 20, 40, and 60 Mg ha^−1^, respectively.

**Figure 2:**
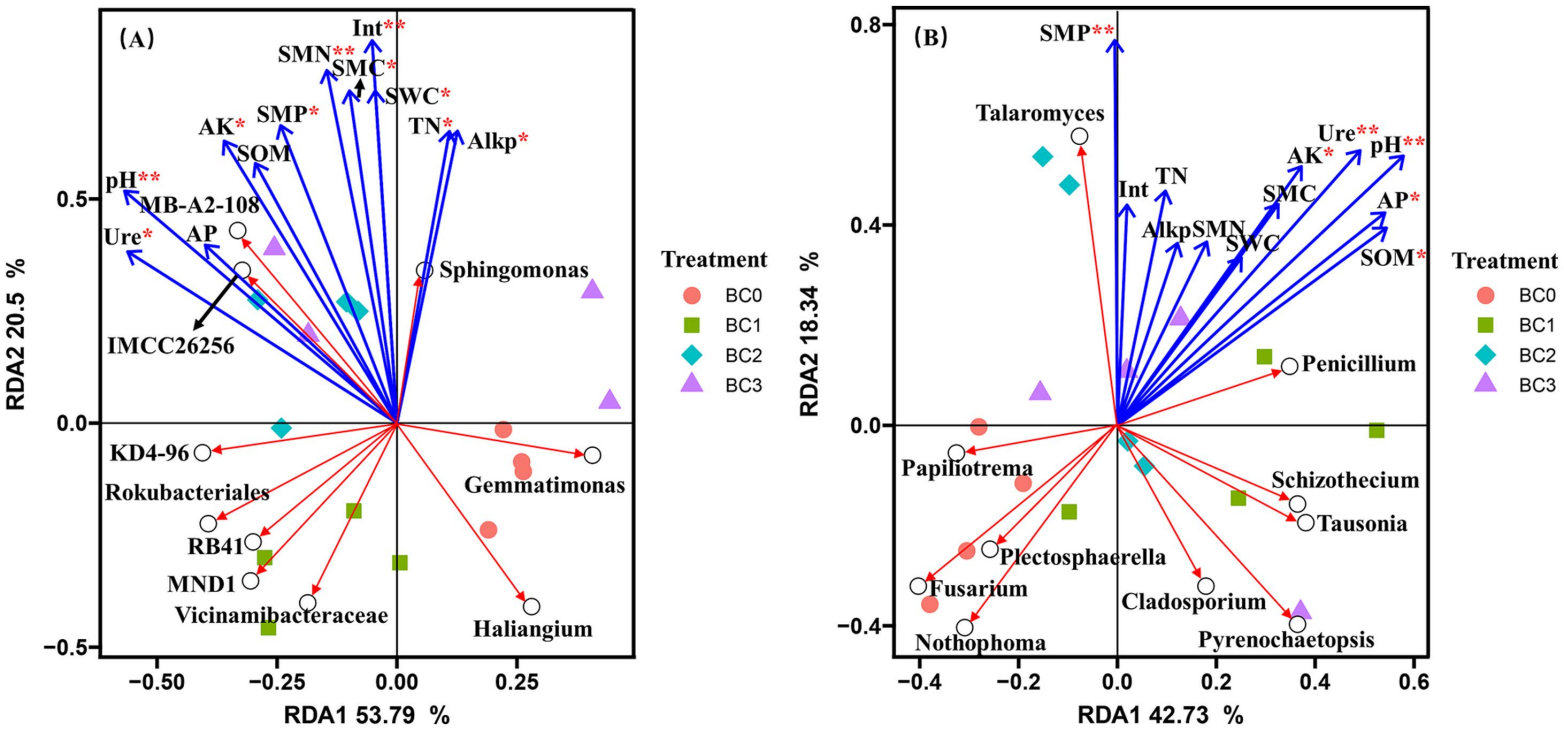
Principal coordinate analysis (PCoA) of bacterial **(A)** and fungal **(B)** communities in different BC treatments based on the Bray–Curtis distances. BC0, BC1, BC2, and BC3 represent BC application rates at 0, 20, 40, and 60 Mg ha^−1^, respectively.

Four years post-application of biochar at varying rates, no notable differences were found in the α-diversity of the soil bacterial community, as measured by the Chao1, Observed species, Shannon, and Simpson indices (Figure 1A). Biochar addition notably decreased the α-diversity of the soil fungal community, with the exception of the Simpson index. Additionally, no significant differences were observed between the BC1, BC2, and BC3 treatments (Figure 1B). PCoA1 and PCoA2 collectively explained 23.6 and 30.8% of the total variance in bacterial and fungal β-diversity, respectively, across all treatments (Figure 2). All four treatments exhibited statistically significant differences from each other (*p* < 0.05) (Table 2).

**Table 2:** PERMANOVA analysis for the relative bacterial abundance of bacteria and fungi.

PERMANOVA	*p* value	
	Bacteria	Fungi
BC0 vs. BC1	0.025	0.033
BC0 vs. BC2	0.028	0.037
BC0 vs. BC3	0.029	0.028
BC1 vs. BC2	0.032	0.031
BC1 vs. BC3	0.032	0.022
BC2 vs. BC3	0.021	0.024

### 3.3 Shifts of soil bacterial and fungal community composition

**Figure 3:**
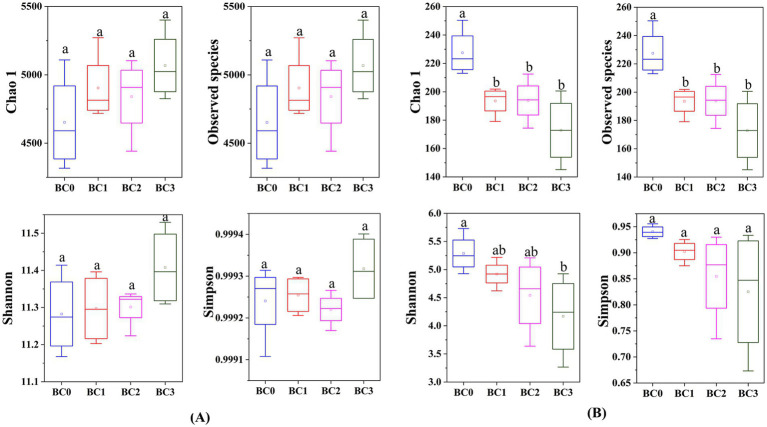
Bacterial **(A)** and fungal **(B)** relative abundance at the phylum and genus levels. BC0, BC1, BC2, and BC3 represent BC application rates at 0, 20, 40, and 60 Mg ha^−1^, respectively.

As illustrated in Figure 3A, soil bacterial communities were dominated by *Proteobacteria* (24.33–25.83%, averaging at 25.03%), *Acidobacteriota* (19.30–21.08%, averaging at 20.29%), *Actinobacteriota* (13.96–17.57%, averaging at 15.24%), *Gemmatimonadota* (10.55–12.55%, averaging at 11.55%), and *Chloroflexi* (8.05–8.93%, averaging at 8.65%). The relative abundance of *Gemmatimonadota*, *Bacteroidota*, and *Myxococcota* was significantly decreased by an average of 10.62, 24.83 and 17.89%, respectively, compared to the control, especially in BC2 treatment (*p* < 0.05) (Supplementary Figure S1). Biochar application notably increased the relative abundances of *Methylomirabilota*, *Actinobacteriota*, and *Planctomycetota* by up to 52.14, 25.87, and 45.19%, respectively, relative to the control (BC0) (*p* < 0.05) (Supplementary Figure S1A). At the genus level, the relative abundances of *MND1*, *MB-A2-108*, *Rokubacteriales*, and *IMCC26256* were notably increased following biochar application (Supplementary Figure S1B). The highest increases were observed at different biochar application rates: *MND1* and *Rokubacteriales* exhibited increases of 46.28 and 56.11% in the BC1 treatment, respectively, while *MB-A2-108* and *IMCC26256* showed increases of 119.31 and 39.70% in the BC2 treatment, respectively, compared to BC0. Conversely, the relative abundances of *Gemmatimonas* and *Haliangium* decreased following biochar addition (Supplementary Figure S1B), with the lowest values occurring in the BC2 treatment, representing reductions of 41.59 and 30.81% compared to BC0, respectively.

Soil fungal communities were dominated by *Ascomycota* (53.89–66.15%, averaging at 58.40%), *Basidiomycota* (18.70–33.14%, averaging at 24.36%), *Rozellomycota* (4.11–19.13%, averaging at 9.93%), and *Mortierellomycota* (2.23–5.72%, averaging at 3.83%) (Figure 3B). Among the phyla, only *Basidiomycota* and *Aphelidiomycota* demonstrated an increase in relative abundance in the BC1 and BC3 treatments, respectively, compared to BC0 (*p* < 0.05) (Supplementary Figure S2A). At genus level, the relative abundance of *Tausonia*, *Schizothecium*, and *Penicillium* significantly increased exclusively in BC1 treatment (Supplementary Figure S2B), exhibiting 1.31, 2.03-, and 107.21-times higher values than BC0, respectively. The BC2 treatment resulted in a 17.29-fold increase in the relative abundance of *Talaromyces* compared to BC0. In contrast, biochar amendments significantly reduced the relative abundances of *Fusarium* and *Nothophoma* in the soil (Supplementary Figure S2B), averaging 54.79 and 70.34% lower than the control, respectively (*p* < 0.05).

### 3.4 Correlations between bacterial and fungal communities and soil variables

**Figure 4:**
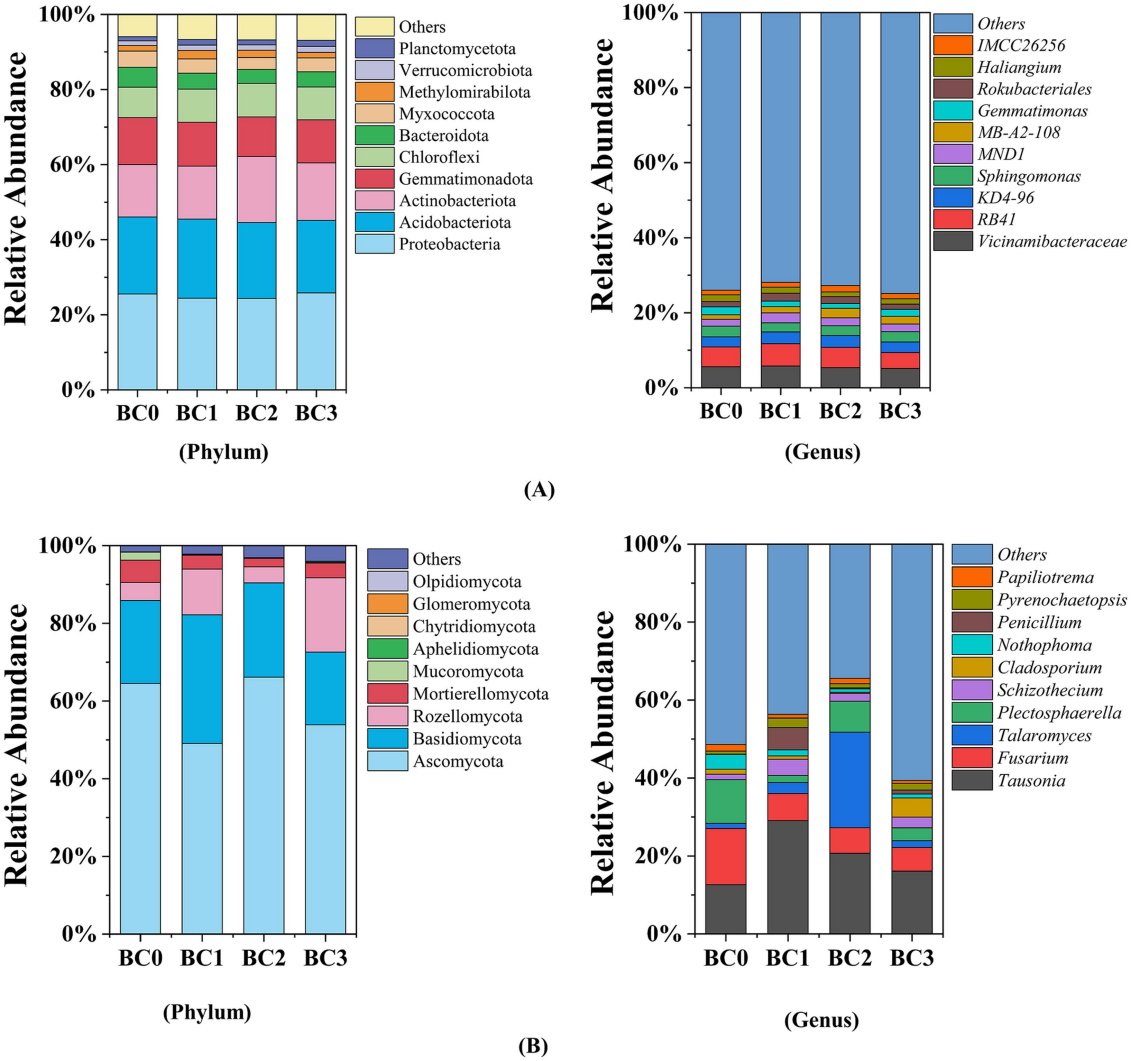
Redundancy analyses (RDA) of the correlations between soil properties (Blue arrows) and the composition of bacterial **(A)**, and fungal **(B)** communities at genus level (Red arrows). *, **, and *** indicate significant differences at 0.05, 0.01, and 0.001 probability levels, respectively. BC0, BC1, BC2, and BC3 represent BC application rates at 0, 20, 40, and 60 Mg ha^−1^, respectively.

Redundancy analysis (RDA) was performed to elucidate the impact of variations in soil physicochemical and microbiological properties on the composition of microbial communities at the genus level. For the bacterial community, RDA1 accounted for 53.79% of the total variation, while RDA2 explained an additional 20.5% of the variation (Figure 4A). The bacterial community composition among four treatments was clearly separated from each other, except for BC2 and BC3 treatment, which were clustered together. pH, SMN, and invertase (*p* < 0.01) were significantly affected bacterial community composition. It also showed that pH, SMN, and invertase were significantly and positively correlated to the genera *IMCC26256, MB-A2-108*, and *Sphingomonas*, while negatively correlated with *Haliangium*. For the fungal community, the major soil properties driving fungal community composition were soil pH, SMP, and urease (*p* < 0.01), and significantly negatively correlated to *Fusarium* and *Nothophoma*, while significantly positively correlated to *Talaromyces* and *Penicillium* (Figure 4B).

### 3.5 Relationships between microbial community diversity, composition, and environmental variables

**Figure 5:**
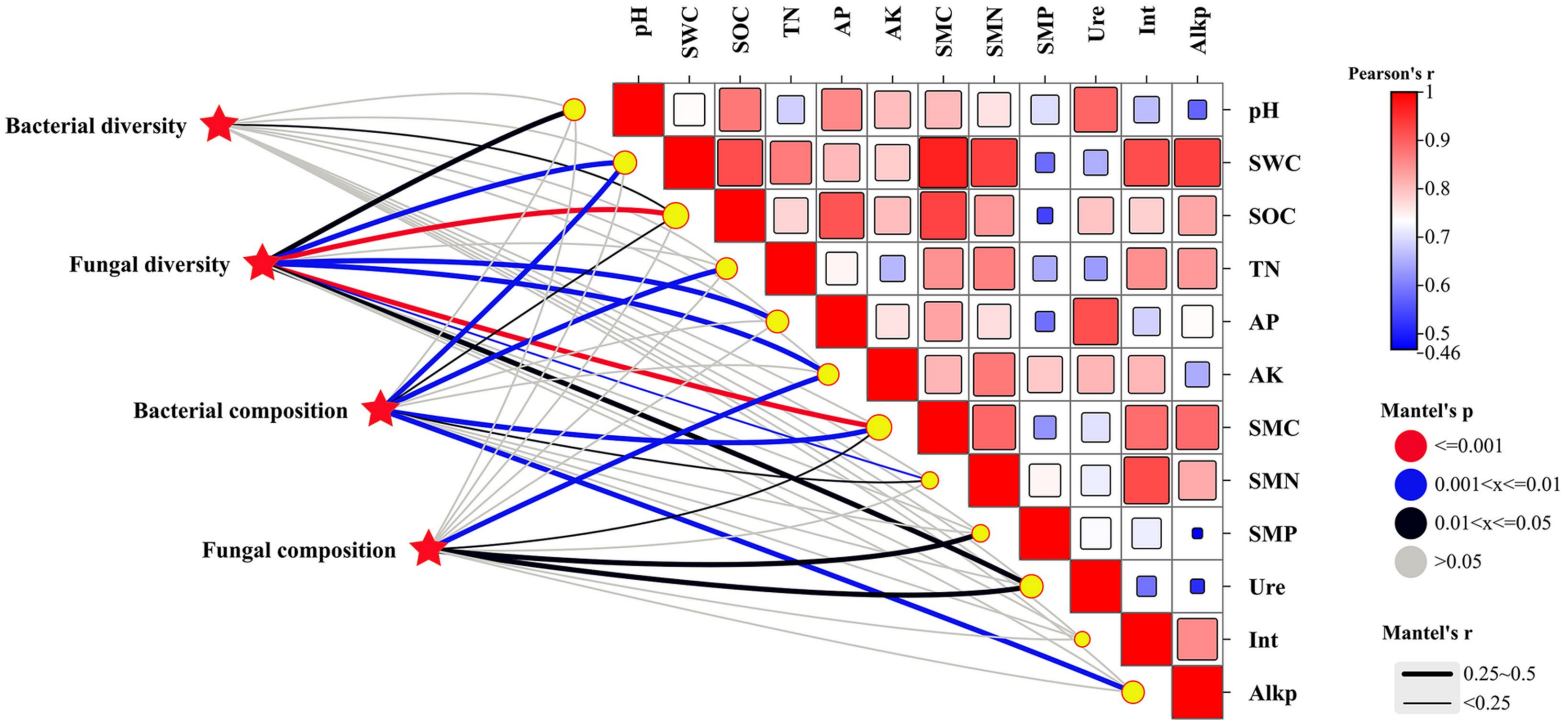
Mantel correlations between environmental factors and taxonomy (genus level) composition of the microbiota and between environmental factors and α-diversity after BC addition. Gray lines indicated no strong Mantel correlation, while black, blue, and red lines indicated a strong Mantel correlation at 0.01 < *p* ≤ 0.05, 0.001 < *p* ≤ 0.01, and *p* ≤ 0.001 levels, respectively.

The diversity and composition of soil microbial communities were favorably affected by soil physicochemical and microbiological properties following the addition of biochar (Figure 5). Bacterial diversity showed a positive association with SOC (*p* < 0.05), while fungal diversity demonstrated positive relationships with SOC, SMC (*p* < 0.001), SWC, AP, AK (*p* < 0.01), pH, and urease activity (*p* < 0.05). Bacterial composition was positively linked to SWC, TN, SMC, and alkaline phosphatase activity (*p* < 0.01). In contrast, fungal composition was significantly associated with AK (*p* < 0.01), SMP, SMC, and urease activity (*p* < 0.05).

## 4 Discussion

### 4.1 Changes in soil properties with biochar addition

Many studies have highlighted that biochar application leads to substantial changes in soil physicochemical properties, including pH and nutrient availability, as well as in microbial characteristics such as biomass and enzyme activity (Lehmann et al., 2011; Doan et al., 2015; Wang et al., 2018; Sun et al., 2021). These effects are modulated by the specific properties of the biochar, such as its feedstock origin and particle size, along with the rates at which it is applied (Zhou et al., 2024). For example, some studies have demonstrated that the application of alkaline biochar significantly increases the soil pH (Liu and Zhang, 2012; Gunarathne et al., 2020). In present study, soil pH showed a significant increase with higher biochar application rates after 4 years, a finding similar to that of Wang et al. (2018), who observed a notable increase in soil pH after 4 years of applying wheat straw biochar. Biochar raises soil pH primarily due to its alkaline properties, which neutralize soil acidity, and the presence of exchangeable base cations such as calcium, magnesium, potassium, and sodium, which contribute to increased soil alkalinity. These cations displace acidic cations, such as hydrogen and aluminum, thereby reducing soil acidity and raising the pH (Van Zwieten et al., 2010). However, no significant difference in soil pH was observed between the BC2 and BC3 treatments. This plateau effect suggests that a threshold exists, beyond which additional biochar does not lead to further increases in soil pH. This is likely due to the saturation of exchange sites with base cations or the buffering capacity of the soil reaching its maximum. Once exchange sites are fully occupied by base cations, additional biochar cannot further displace acidic cations, causing the pH to stabilize.

The addition of biochar has been shown to enhance soil water retention, primarily through its effects on soil structure and porosity (Yadav et al., 2018). In the present study, biochar application resulted in a substantial enhancement of SWC in aeolian sandy soil, with a positive correlation observed between SWC and increasing biochar application rates. Similarly, Razzaghi et al. conducted a meta-analysis that assessed the impact of biochar on soil water retention, revealing that biochar notably increased the water retention capacity of soils, especially in coarse-textured soils (Razzaghi et al., 2020). The increased SWC is likely due to the decrease in soil bulk density and the improvement in soil aggregation after biochar application, which collectively enhance the soil’s capacity to retain water. These results highlight biochar’s vital function as a soil amendment in improving water retention, thereby contributing to sustainable agriculture, particularly in arid regions.

Many studies have found that biochar application enhances soil fertility, particularly by increasing SOC, TN, and the availability of key nutrients (Van Zwieten et al., 2010; Zhang L. et al., 2021). Typically, biochar contains a substantial amount of recalcitrant and aromatic carbon, which is resistant to decomposition and contributes to increased soil carbon content (Che et al., 2024). In this study, the addition of biochar led to a 6.95–14.13% increase in SOC content over a four-year period. However, these responses are closely influenced by factors such as the biochar application rate and soil type, which can affect the extent of SOC increase. For example, Dong et al. (2019) observed that after 5 years of biochar application, soil organic matter (SOM) increased by 18.8, 42.4, and 62.3% compared to the control, with application rates of 30, 60, and 90 Mg ha^−1^, respectively. Zhang et al. (2023) also demonstrated that 5 years after biochar application, SOC in sandy soil significantly increased with higher biochar application rates. In our study, SOC enhancement exhibited a positive correlation with biochar application rates, resulting in increases of 6.95, 9.42, and 14.13% in BC1, BC2, and BC3, respectively, compared to BC0 (Table 1). This finding supports the long-lasting impact of biochar in enhancing SOC content, a key factor for sustainable soil management, especially in arid and semi-arid sandy soils, which often face challenges such as low organic matter content and poor water retention.

In this study, biochar application to aeolian sandy soils led to significant increases in soil TN, AP, and AK content. These changes can be primarily attributed to two key factors: (i) biochar facilitated the release of soil nutrients, and (ii) it affected soil microbial diversity and abundance, especially microorganisms involved in the nitrogen, phosphorus, and potassium cycles, thereby enhancing the soil environment (Singh et al., 2022). Significant improvements in soil available nutrients were noted in the treatments with higher biochar application rates by the fourth year after biochar addition. This increase is likely due to the gradual release of nutrients from the biochar over time (Zhou et al., 2024).

Soil microbial biomass and enzymatic activity are considered key indicators of soil quality, providing insights into changes in fertility, nutrient availability, and overall soil health. In this study, significant enhancements were observed in soil microbial biomass (C, N, and P), as well as in the activities of three soil extracellular enzymes following the addition of biochar, particularly at higher biochar application rates. Similar beneficial effects of biochar on soil microbial biomass and enzymatic activity have been reported in other studies (Zheng et al., 2016; Li et al., 2023). The underlying mechanism can be attributed to three primary aspects. First, biochar, owing to its highly porous structure and large surface area, offers abundant adsorption sites and creates a favorable microhabitat for various soil microorganisms—including bacteria, mycorrhizal fungi, and actinomycetes—thereby promoting their growth and reproduction, and potentially enhancing the microbial assimilation of biochar itself (Zheng et al., 2025). This, in turn, enhances soil microbial biomass and stimulates the secretion and activity of the enzymes under investigation (Lehmann et al., 2011). Second, incorporating biochar significantly increases SOC content, thereby supplying more substrates, including labile organic carbon, for soil enzymes (Dai et al., 2021). Finally, biochar addition enhances crop root development, which stimulates rhizodeposition, fostering microbial growth and boosting the secretion and activity of the enzymes examined (Akumuntu et al., 2024). In this study, lower rates of biochar application, even after 4 years, did not produce significant changes in the majority of microbiological parameters, including SMN, SMP, urease, invertase, and alkaline phosphatase activity (Table 1). However, higher rates of biochar consistently exhibited greater increases in soil microbial biomass content and the activities of urease, invertase, and alkaline phosphatase, even after 4 years of application. This finding suggests that the impact of biochar on soil microbiological parameters is strongly dependent on the rate of application. Lower application rates of biochar may not significantly alter soil physicochemical properties over an extended period following its application, possibly due to insufficient alteration of soil structure or inadequate provision of substrates and habitats for soil microorganisms. Conversely, higher rates of biochar application can result in substantial long-term benefits for soil health, attributable to its prolonged persistence and greater improvements in soil physicochemical and microbiological properties.

### 4.2 Changes of bacterial and fungal community diversity and composition to biochar addition

In this study, the addition of biochar generally decreased fungal diversity, whereas bacterial diversity remained unchanged compared to the control. In a similar study, Zhang et al. observed that biochar application at rates of 10, 30, and 50 Mg ha^−1^ led to a significant reduction in the diversity of both bacterial and fungal communities in black soil, as demonstrated by a three-year field trial in northeastern China, compared to the control (Zhang L. et al., 2021). Zheng et al. (2016) found that, 4 years following a one-time application of biochar at rates of 20 and 40 Mg ha^−1^, bacterial α-diversity significantly increased, while fungal α-diversity decreased, in sandy loam soils of southwest China. However, Fan S. et al. (2020) found that a single application of biochar after 6 years at rates of 4, 8, and 12% (w/w) significantly increased soil bacterial diversity compared to the control, while no significant variations in bacterial diversity were detected across soils treated with different biochar application rates. Moreover, Hu et al. reported that, after 7 years, the addition of biochar at rates of 15.75, 31.50, 63.00, and 126.00 Mg ha^−1^ had no statistically significant effect on the microbial α-diversity compared to the unamended soil (Hu et al., 2023). In contrast, Zheng et al. found that equal amounts of rapeseed straw biochar and rice straw biochar significantly increased the bacterial Shannon and Simpson indices, and also enhanced the fungal Simpson index compared to the control (Zheng et al., 2025).

These findings suggest that the response of bacterial and fungal community diversity to biochar amendment varies and is influenced by multiple factors such as application rates, soil types, and experimental durations.

In this study, the predominant bacterial phyla were in orders of *Proteobacteria, Acidobacteriota, Actinobacteriota, Gemmatimonadota*, and *Chloroflexi*. Compared to CK, the relative abundance of *Actinobacteria, Methylomirabilota,* and *Planctomycetota* increased in the soil amended with biochar. The phylum *Actinobacteria* is essential for breaking down complex, resistant polymers like cellulose and chitin (Ren et al., 2020). In this study, the application of biochar increased the abundance of *Actinobacteria*, likely due to the introduction of recalcitrant carbon sources by the biochar, which stimulated the growth of these microorganisms (Hu et al., 2023). Among these, *Methylomirabilota* and *Planctomycetota* exhibited the most pronounced response, with relative abundances increasing by 52.14 and 45.19%, respectively, in response to biochar treatments compared to the control (Supplementary Figure S1A). *Methylomirabilota* is involved in nitrogen cycling (Zhang X. et al., 2021), while *Planctomycetota* are known for their roles in carbon fixation and specific nitrogen cycling processes, such as anammox (Hug et al., 2013). This suggests that biochar amendment may create favorable conditions for these bacteria, potentially enhancing both nitrogen cycling processes and carbon fixation. Notably, in our study, we observed a significant reduction in the relative abundance of Bacteroidetes, ranging from 19.98 to 30.24%, especially at the higher biochar application rates (BC2 and BC3). This reduction may be attributed to intensified competition for limited nutrients, which may lead to *Bacteroidetes* being outcompeted by other microbial groups that are better adapted to these low-nutrient conditions.

The fungal community composition across all four treatments was similar, with *Ascomycota, Basidiomycota*, and *Rozellomycota* being the dominant phyla (Figure 3B). This observation is consistent with previous studies conducted in agricultural soils (Xu et al., 2012; Zheng et al., 2016). Biochar amendment primarily influenced soil fungal communities at the genus level, with minimal impact observed at the phylum level. Notably, the relative abundance of *Fusarium* and *Nothophoma* was significantly reduced by 52.03–57.97% and 59.02–77.41%, respectively, in biochar-amended treatments compared to non-amended soils (Supplementary Figure S2B). Similar findings were reported by Yao et al. (2017) who observed a significant decrease in the relative abundance of Fusarium following a three-year application of biochar at a rate of 200 Mg ha^−1^ in black soil. Similarly, Ahmad et al. observed a significant decline in the relative abundance of the soil pathogen *Fusarium* after applying 15 Mg ha^−1^ of palm biochar (Ahmad et al., 2024). Additionally, studies by Matsubara et al. (2002) and Elmer and Pignatello (2011) demonstrated that coconut biochar and hardwood-dust biochar were effective in suppressing *Fusarium* infection on asparagus roots. As we known, the genera *Fusarium* and *Nothophoma* are extensive and taxonomically complex, comprising numerous species that act as crop pathogens, leading to a wide array of plant diseases, including wilts, rots, and blights (Summerell et al., 2010; Zhang X. et al., 2021). The underlying mechanisms may include the following: First, biochar-induced soil microorganisms competing with pathogens for carbon, producing harmful compounds, or parasitizing the pathogens (Dai et al., 2018). Secondly, biochar contributes to the enhancement of plant systemic resistance by facilitating improved nutrient retention and availability, promoting increased root biomass, depth, and branching, and fostering a more conducive environment for arbuscular mycorrhizal colonization through the amelioration of soil structure and nutrient dynamics (Dai et al., 2021). This suggests the addition of BC appears to create a more balanced and healthy soil environment by suppressing harmful fungi and promoting beneficial soil properties. Furthermore, the application of BC led to an increased abundance of certain fungal genera, including *Tausonia, Talaromyces, Schizothecium,* and *Penicillium*, but their abundance varies in BC-amended soils. Numerous studies have demonstrated that BC application increased the relative abundance of *Tausonia* (Liu H. Y. et al., 2020), *Talaromyces* (Idbella et al., 2024), *Schizothecium* (Guo et al., 2024), and *Penicillium* (Zheng et al., 2016). These genera are crucial for soil organic matter decomposition and nitrogen cycling, which improve nutrient cycling and availability, thereby enhancing soil fertility and supporting the long-term sustainability of agricultural systems.

### 4.3 Relationship between microbial communities and soil properties

pH is a principal determinant influencing the composition of soil microbiota, both at local and global scales (Delgado-Baquerizo et al., 2018). Previous studies have shown that neutral to slightly alkaline pH levels typically favor bacterial growth while inhibiting fungal development (Chen et al., 2013). In our research, the biochar was alkaline, and its application significantly influenced fungal diversity, whereas it had no substantial effect on bacterial diversity (Figure 5). This observation can be attributed to differences in pH tolerance, nutrient requirements, and habitat preferences between bacteria and fungi. It indicates that biochar may selectively favor bacterial communities in alkaline environments, potentially leading to long-term changes in soil microbial community structure and function.

The diversity of the bacterial communities showed no strong relationships with all tested soil properties, while eight soil properties were found to have a strong influence on the diversity of fungal communities (Figure 5). Similarly, Liu et al. highlighted that compared with bacterial diversity, bacterial community composition was more sensitive and responds more quickly to a variety of soil physicochemical properties (Liu F. et al., 2020). These results suggest that fungal communities are more sensitive to soil conditions compared to bacterial communities. However, soil properties had a substantial impact on the composition of both bacterial and fungal communities. This observation implies that while soil properties may not greatly affect the overall diversity of bacterial communities, they can shape the specific bacterial species present. These findings emphasize the intricate relationship between soil properties and microbial communities, underscoring the necessity for focused research to elucidate the factors that govern microbial community structure and functionality within soil ecosystems.

Although this study was conducted at a single site in a semi-arid region under a buckwheat cropping system, the observed microbial and nutrient responses to biochar application may have broader implications for other coarse-textured or degraded soils in similar climatic conditions. Nevertheless, these effects are likely to be modulated by site-specific factors such as native soil characteristics, cropping systems, and the properties of the applied biochar, which are, in turn, influenced by feedstock type and pyrolysis conditions (Siddiqui, 2025). In this study, the biochar was produced from maize straw at 450°C, exhibiting distinct physicochemical characteristics that may differ substantially from those of other biochars, thereby necessitating caution when extrapolating the results. Moreover, as the measurements were taken 4 years after a single application, temporal variability in soil microbial dynamics and nutrient cycling likely contributed to the observed patterns. Taken together, these considerations highlight the need for further research across multiple sites, agroecosystems, and time scales to better understand the long-term persistence and broader applicability of biochar amendments.

While significant correlations were identified between microbial community composition and soil C and N cycling parameters, the underlying functional mechanisms remain to be elucidated. Future studies incorporating diverse biochar types, agroecosystems, and functional omics approaches—such as metagenomics or transcriptomics—are needed to identify key functional genes and metabolic pathways driving microbial responses. Such integrative approaches will enhance our understanding of the functional linkages between soil microbiota and nutrient cycling processes, particularly under long-term field conditions.

## 5 Conclusion

In the aeolian sandy soils of Inner Mongolia, biochar application has been shown to notably improve soil fertility and modify the composition of bacterial and fungal communities over a four-year period. Notably, these effects are more pronounced with higher BC application rates. The fungal community showed a stronger response to biochar application than the bacterial community, especially at the genus level. Additionally, biochar treatment significantly modified the bacterial composition, notably enhancing the relative abundance of genera associated with nitrogen cycling and carbon fixation. The addition of BC primarily changed fungal community composition mainly by reducing the relative abundances of potential soil-borne plant pathogens, such as *Fusarium* and *Nothophoma*. The composition of bacterial communities was mainly shaped by pH, SMN, and invertase activity, whereas pH, SMP, and urease activity played a key role in influencing the fungal community structure. These microbial changes are linked to improved soil chemical properties and enzyme activities, highlighting biochar’s multifunctional role in enhancing soil structure, nutrient availability, and microbial balance to promote nutrient cycling and suppress pathogens.

Our study highlights the significant role of BC in enhancing the fertility of aeolian sandy soils, leading to a more balanced and healthier soil environment 4 years post-application, with pronounced effects observed at higher application rates. Despite its potential as an effective soil amendment for aeolian sandy soils, further studies are needed to explore the long-term effects of BC on soil quality, carbon sequestration, greenhouse gas emissions, and the diversity and functionality of carbon and nitrogen cycling-related gene communities in semi-arid regions.

## Data Availability

The datasets presented in this study can be found in online repositories. The names of the repository/repositories and accession number(s) can be found in the article/Supplementary material.

## Footnotes

^1^ https://docs.qiime2.org/2021.8/tutorials/

^2^ https://cran.dcc.uchile.cl/
