# Supplementary material for: Four-year residual impacts of single biochar application on soil fertility and microbial community structure in aeolian sandy soils of semi-arid Inner Mongolia
Source: Front Microbiol. 2025 Jul 16;16:1619992. doi: 10.3389/fmicb.2025.1619992 (PMC12307304; doi:10.3389/fmicb.2025.1619992)
Supplement: Supplementary file 1 [file Data_Sheet_1.docx]

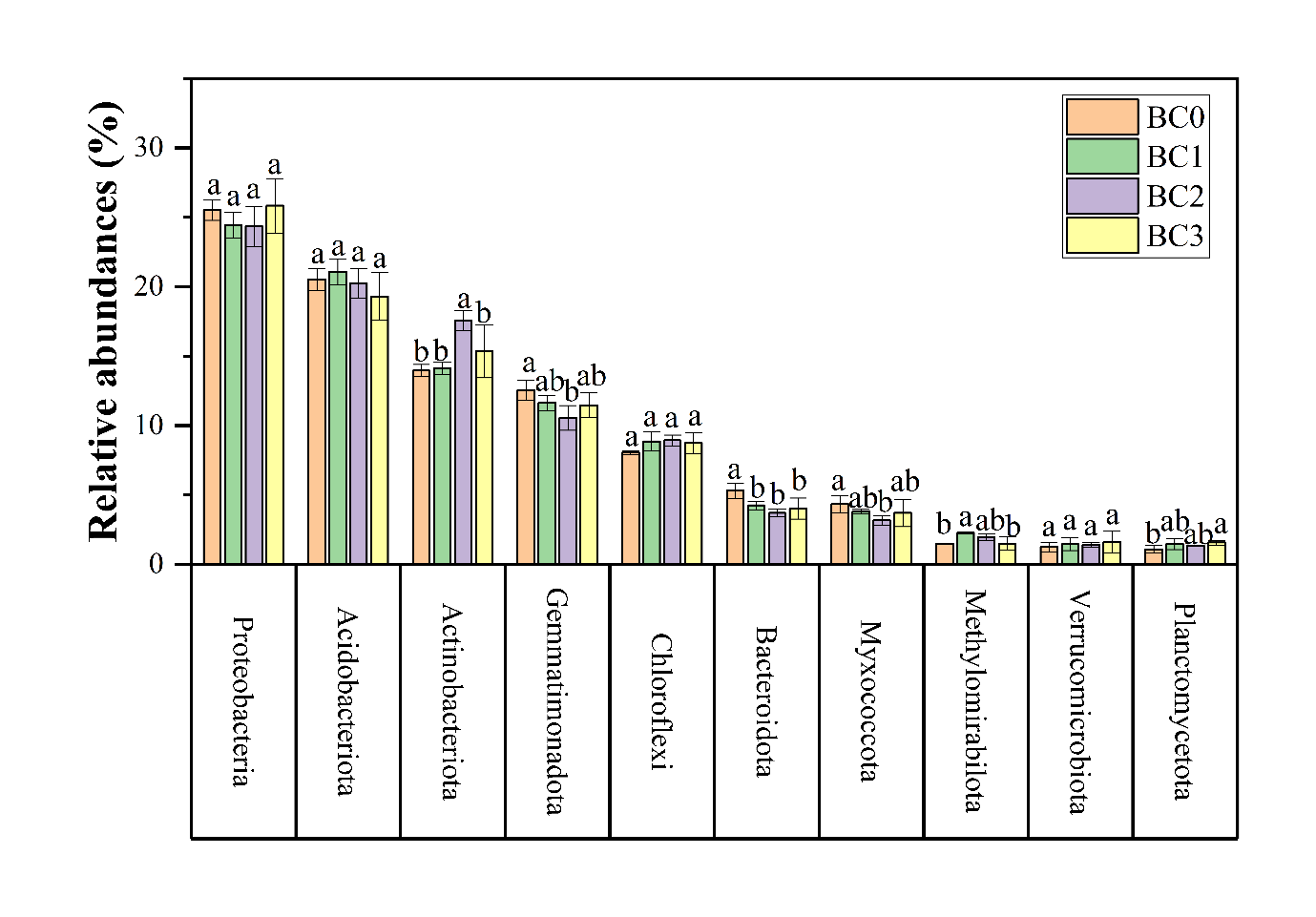
**Figure S1** Relative abundance of the 10 most abundant phyla (A) and genera (B) of bacteria (> 1%) across all treatments. Vertical bars are standard errors of the mean (n = 4). Pairs of bars with the same letter between treatments within the same phylum and genus are not significantly different at *p* ≤ 0.05. BC0, BC1, BC2, and BC3 represent BC application rates at 0, 20, 40, and 60 Mg ha^-1^, respectively.

A)


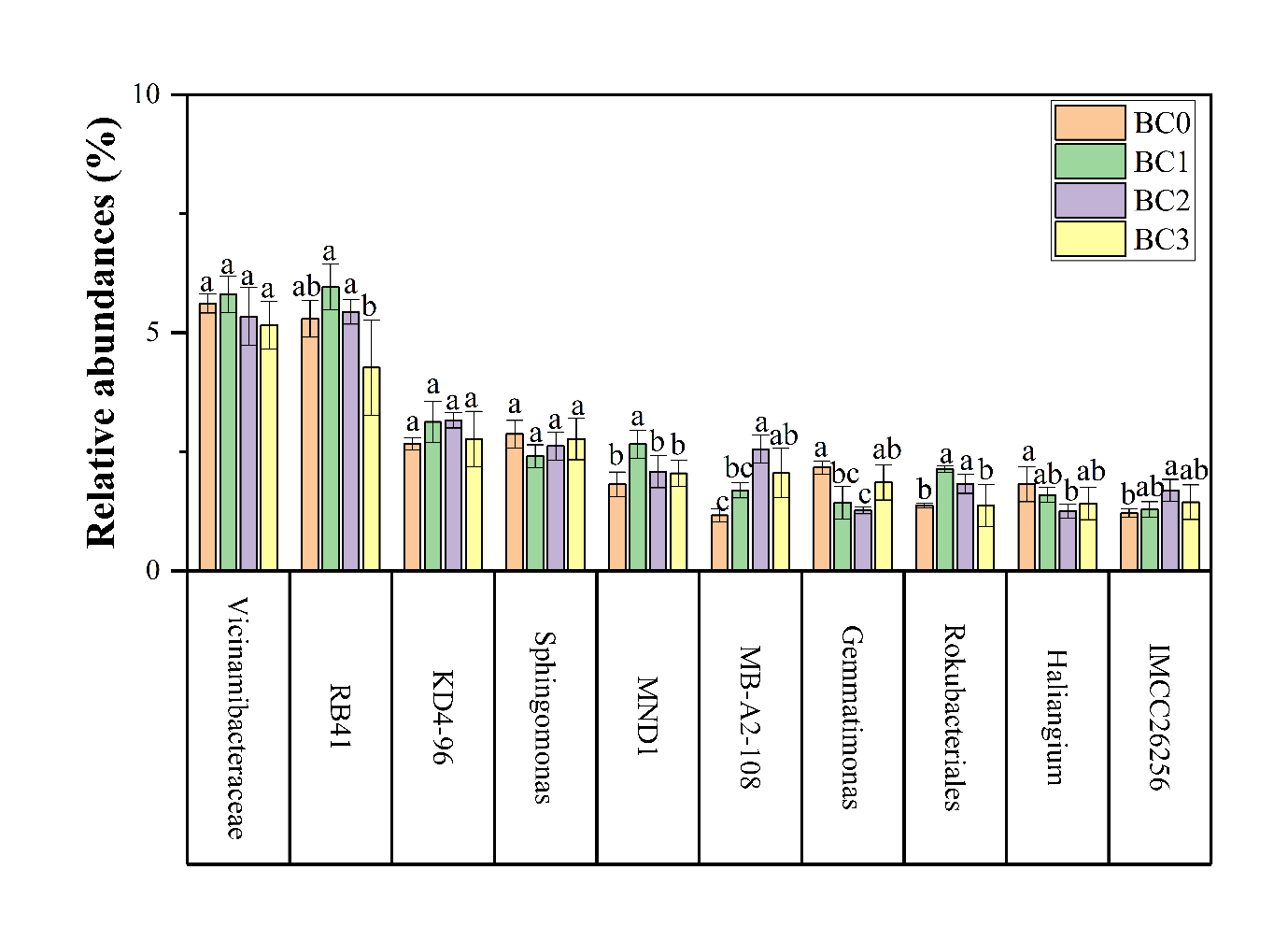


B)

**Figure S2** Relative abundance of the 4 most abundant phyla (A) and the 10 most abundant genera (B) of fungi (> 1%) across all treatments. Vertical bars are standard errors of the mean (n = 4). Pairs of bars with the same letter between treatments within the same phylum and genus are not significantly different at *p*≤ 0.05. BC0, BC1, BC2, and BC3 represent BC application rates at 0, 20, 40, and 60 Mg ha^-1^, respectively.


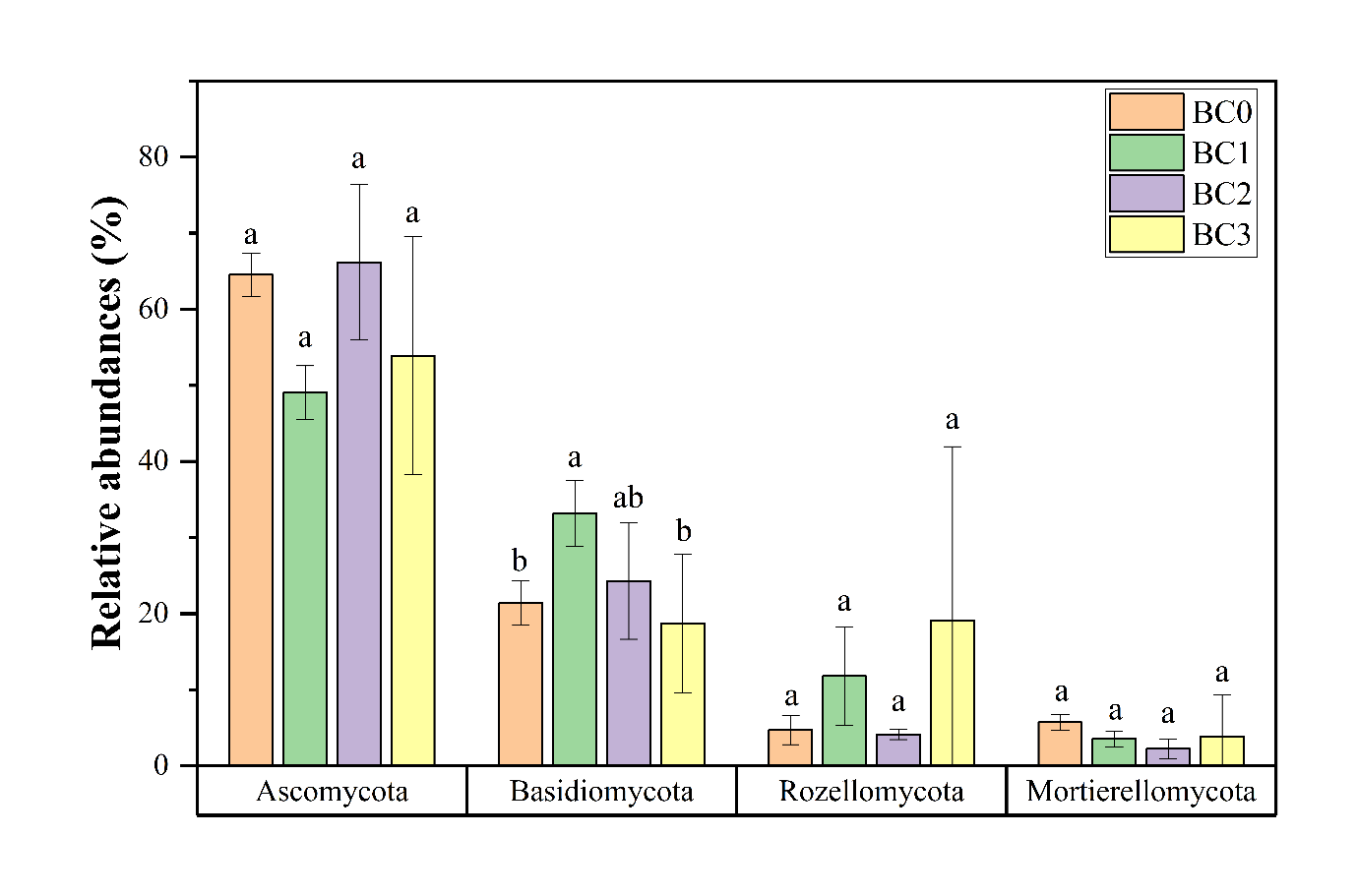


A)


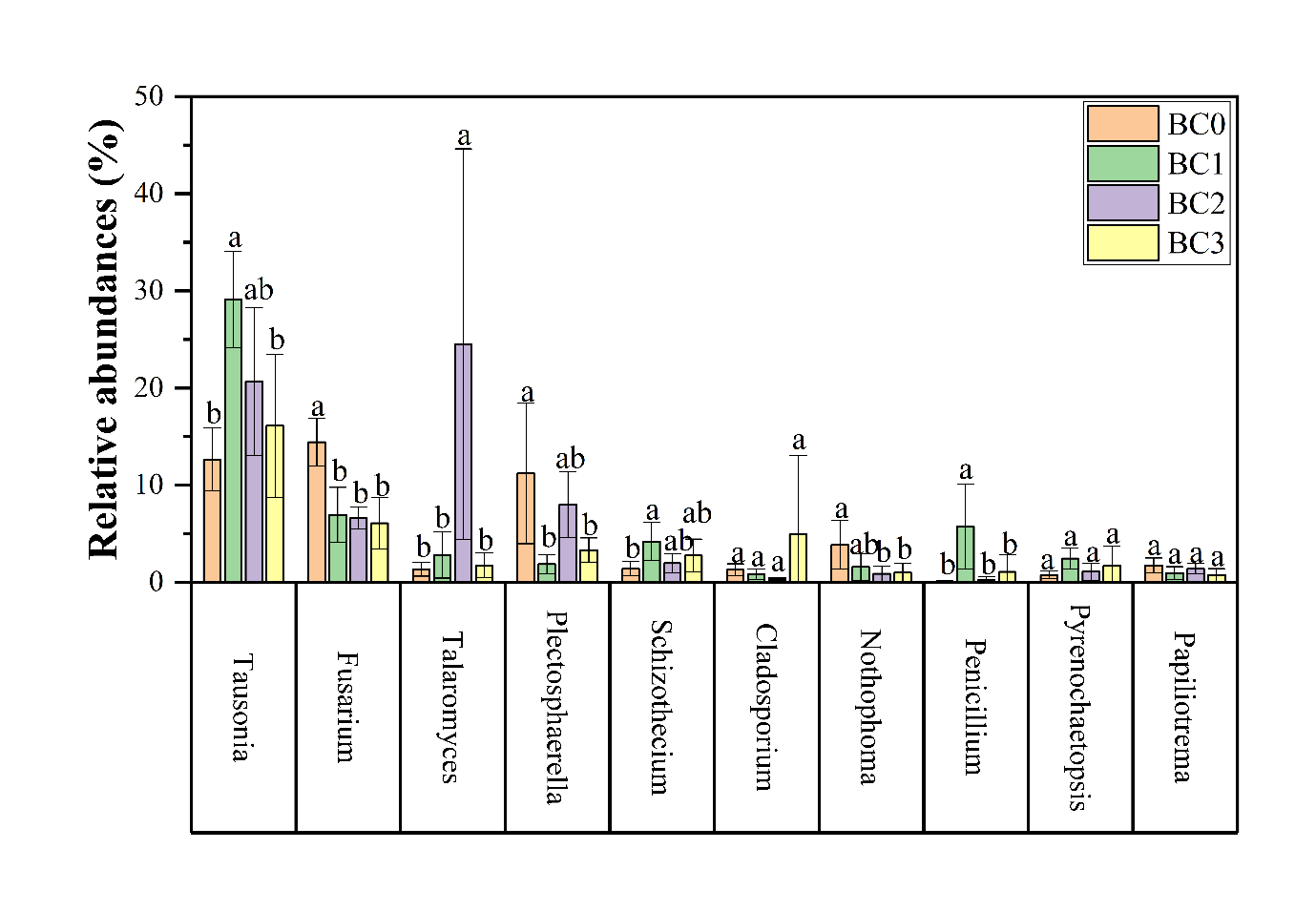


B)
